# Alveolar distribution of nebulized solution in health and lung injury assessed by confocal microscopy

**DOI:** 10.14814/phy2.70018

**Published:** 2024-10-25

**Authors:** Zahra Ansari, John Battikha, Charul Singh, Carrie E. Perlman

**Affiliations:** ^1^ Department of Biomedical Engineering Stevens Institute of Technology Hoboken New Jersey USA

**Keywords:** confocal microscopy, lung injury, nebulization, parenchymal distribution

## Abstract

Parenchymal distribution of nebulized drug in healthy and diseased lungs has not, as evident from a literature review, been well characterized. We use a vibrating mesh nebulizer to deliver fluorescein solution in vivo to healthy or intratracheal‐lipopolysaccharide (LPS)‐instilled anesthetized rats in dorsal recumbency, or ex vivo to the lungs of LPS‐instilled rats. Following in vivo nebulization (healthy/LPS‐instilled), we quantify fluorescein intensity distribution by confocal microscopy in standard locations on the surface of freshly isolated lungs. Following LPS instillation (in vivo/ex vivo nebulization), we quantify fluorescein intensity in visibly injured locations. In standard locations, there is uniform, low‐intensity basal fluorescein deposition. Focal regions receive high deposition that is, in upper (cranial), middle, and lower (caudal) locations, 6.4 ± 4.9, 3.3 ± 3.0, and 2.3 ± 2.8 times greater, respectively, than average basal intensity. Following LPS instillation, deposition in moderately injured regions can be high or low; deposition in severely injured regions is low. Further, actively phagocytic cells are observed in healthy and LPS‐instilled lungs. And LPS particularly impairs mechanics and activates phagocytic cells in the male sex. We conclude that a low level of nebulized drug can be distributed across the parenchyma excepting to severely injured regions.

## 1 INTRODUCTION

Effective and efficient delivery of a nebulized substance involves targeting appropriate airway generations and distributing the substance homogenously across lobes and segments. Nebulization is used to treat airway diseases such as asthma, chronic obstructive pulmonary disease, and cystic fibrosis (Albertson et al., 2015; Ratjen et al., 2019; Tashkin, 2016). Nebulization to the parenchyma is also of interest and has been tested for delivery of pulmonary therapeutics such as antibiotics and surfactant (Cummings et al., 2020; Monsel et al., 2021) and of the systemic therapeutic insulin (Pittas et al., 2015). Innovations in aerosol science have markedly improved targeting and distribution capabilities (Longest et al., 2019). However quantification of nebulized substance deposition pattern, particularly at the parenchymal level and under disease conditions, is lacking.

In murine studies, multiple approaches have been used to quantify distribution of nebulized solution between airway generations or lung lobes. One approach is to homogenize tissue and extract and analyze a nebulized marker, for example spectrophotometric absorbance or luminescence of a dye (Hofstetter et al., 2004; Köping‐Höggård et al., 2005; MacLoughlin et al., 2009; Tronde et al., 2002). In this case, quantification of spatial distribution across tissue is not possible. Other approaches allow for quantification of spatial distribution. Fluorescent or luminescent markers may be detected with whole‐body or isolated‐lung imaging; in such images, resolution can exceed 10 μm but has generally been markedly lower (Menon et al., 2014; Patel et al., 2019; Refaat et al., 2022; Shi et al., 2021; Urso et al., 2023). Appropriate substances may be detected volumetrically by magnetic resonance imaging (resolution as fine as 0.5–1 mm), positron emission tomography‐computed tomography (resolution ~2 mm), or x‐ray phase contrast imaging (Cossío et al., 2018; Gradl et al., 2019; Oakes et al., 2013; Thomas et al., 1997; Wang et al., 2016). Finally, fixed or frozen tissue sections may be imaged with a high‐resolution technique such as fluorescence microscopy (submicron resolution of a nebulized fluorescent marker) or desorption electrospray ionization‐mass spectrometry imaging (DESI‐MSI; 10–50 μm resolution) (Bäckström et al., 2018; Beng et al., 2021; Desprez et al., 2019; Hamm et al., 2020; Leong et al., 1998; Noverraz et al., 2023). With fluorescence microscopy and DESI‐MSI, detailed quantification of spatial distribution is possible. However, the analysis performed for most studies has tended to be qualitative or to integrate deposition over large regions such that spatial resolution has been lost.

From the above studies, some trends in deposition pattern can be identified. With small ≤2 μm particles delivered by nasal inhalation, deposition tends to vary between lobes (Liu et al., 2016; Thomas et al., 1997); mechanical ventilation through an endotracheal tube can increase deposition uniformity (Hofstetter et al., 2004). With larger 3–6 μm particles delivered by nasal inhalation, distribution across lobes is relatively uniform (Cossío et al., 2018; Eirefelt et al., 1992; Leong et al., 1998; Patel et al., 2019; Wang et al., 2016), excepting in one study in which deposition was greater in the upper than lower lobes (Noverraz et al., 2023). With the same 3–6 μm particle size, mechanical ventilation through an endotracheal tube maintains deposition uniformity across lobes (MacLoughlin et al., 2009; Urso et al., 2023). Very large, 10–20 μm, particles have been delivered by intratracheal nebulization (Cossío et al., 2018; Leong et al., 1998; Tronde et al., 2002). With this delivery method, however, lobar deposition can be variable, sensitive to instrument position or lower in lower lobes (Cossío et al., 2018; Leong et al., 1998).

Beyond lobar deposition, a few groups have attempted to distinguish between intrapulmonary conducting airway and parenchymal deposition of nebulized substances. In two studies, the parenchyma was dissected from the airways, but the airway generation to which the investigators dissected was not specified (Köping‐Höggård et al., 2005; Liu et al., 2016). Beng et al. (2021) obtained frozen, sagital sections 1/4, 1/2, and 3/4 of the way through the left lung and imaged with DESI‐MSI. They analyzed only the middle and outer sections, in which they attributed signal to the terminal bronchioles and parenchyma, respectively. But they used large regions of interest (ROIs) that encompassed >50% of the area of each section and precluded detailed analysis. Backstrom et al. (2018) segmented DESI‐MSI images to distinguish between deposition in airway epithelial, airway sub‐epithelial, and alveolar regions. Following inhalation by rats of jet‐nebulized salmeterol, a long‐acting β2‐adrenoreceptor agonist, they found that signal intensity in the airway regions was >5× that in the alveolar region.

In a detailed analysis, Leong et al. (1998) quantified the fractional areas of airways and parenchymal compartments that received nebulized substance. They exposed rats in a chamber to small, 1.1 μm particles of nebulized ink that stained the airways or nebulized large, 9.0 μm particles of the ink directly into the trachea; sectioned the lungs with a regular and comprehensive scheme that followed the airway tree; and quantified spatial extent of ink delivery in the histologic sections. With chamber inhalation of small particles, all airways, all alveoli in right and left lobes, and 52% of alveoli in the postcaval lobe received dye; low postcaval lobe ventilation has been noted previously (Yen et al., 2019). With intratracheal nebulization of large particles, deposition decreased with increasing airway generation, deposition in the airways was heterogeneous, and whether there was greater deposition in proximal or distal alveoli varied with lung lobe. Beyond this assessment of fractional area of deposition, spatial variation in level of deposition of a nebulized substance in the parenchyma has not been characterized.

Further, though nebulization is intended for disease treatment, nebulized substance distribution has been studied almost exclusively in healthy animals. In an intratracheal bacterial inoculation model, Shi et al. (2021) used a vibrating mesh nebulizer to administer fluorescently‐labeled mesenchymal‐stem‐cell‐derived extracellular vesicles and assessed distribution with low‐resolution fluorescent imaging of the whole body in vivo and of isolated lungs post mortem. Whether nebulized solution was delivered to injured lung regions, however, was not determined.

Vibrating mesh nebulizers, and particularly the Aeroneb nebulizer from Aerogen, are used in many murine studies (Beng et al., 2021; Cossío et al., 2018; Gradl et al., 2019; MacLoughlin et al., 2009; Menon et al., 2014; Noverraz et al., 2023; Patel et al., 2019; Ravikumar et al., 2016; Shi et al., 2021; Urso et al., 2023; Wang et al., 2016; Zhang et al., 2018). Vibrating mesh nebulizers generate relatively large, ~5 μm, aerosol particles. Although this size particle should preferentially deposit in larger airways, there is solution delivered to the alveoli. In the present study we use the Aerogen nebulizer to deliver fluorescent solution to the lungs. And we use confocal microscopic imaging of surface alveoli in freshly isolated lungs to quantify heterogeneity of deposition.

## 2 METHODS

To assess distribution of nebulized solution, we use fluorescein as a fluorescent tracer.

### 2.1 Experimental protocol

We perform this study in Sprague Dawley rats (*n* = 19, 255 ± 35 [SD] g, males/females). Rats are housed in actively‐ventilated cages with controlled temperature, humidity, and light (12 h light/12 h dark) and free access to water and food (5053 PicoLab RodentDiet 20, W.F. Fisher, Somerville, NJ). We perform four sets of experiments: in vivo nebulization in healthy rats, in vivo nebulization in rats that previously received intratracheal instillation of lipopolysaccharide (LPS), ex vivo nebulization in lungs from rats that previously received intratracheal LPS, and healthy un‐nebulized control rats.

#### 2.1.1 In vivo nebulization in healthy rats

We anesthetize a rat (2.5% isoflurane in 100% oxygen), place the rat on a heating pad, targeting a body temperature of 37°C, and cannulate a tail vein for intravenous (IV) injections. We place the rat in dorsal recumbency, perform a tracheotomy, cannulate the trachea, and use a three‐way stopcock to connect the tracheal cannula to both the circuit of a mechanical ventilator (Inspira model 683, Harvard Apparatus, Holliston, MA) and a pressure transducer for recording airway entrance pressure, *P*_*AW*_.

The ventilation circuit is constructed of 1/8‐in inner diameter (ID) × 1/4‐in outer diameter silicone tubing with 4‐in long inspiratory and expiratory paths between the ventilator and the junction of a 0.1‐in ID Y‐connector. There is an additional 4 in of length between the Y‐junction and the distal end of the tracheal cannula. Inserted into the inspiratory limb (included in total 4‐in length) is a T‐adapter (Kent Scientific, Torrington, CT) that holds a vibrating mesh nebulizer (Aeroneb Pro, Kent Scientific) filled with 5 mL of 5 mM fluorescein (MW 332 g/mol; 00298–17, Cole Parmer, Vernon Hills, IL) in normal saline (NS).

We commence ventilation (6 mL/kg tidal volume; 4 cmH_2_O positive end‐expiratory pressure, PEEP; 75 breaths/min; 1:2 inspiratory:expiratory ratio) and, to prevent spontaneous breathing, administer an IV paralytic (0.3 mL of 1 mg/mL pancuronium bromide; P1918, MilliporeSigma, Burlington, MA). We monitor heart rate (HR) and peripheral arterial oxygen saturation, *S*_*P*_ *O*_*2*_, by pulse oximetry (Physiosuite, Kent Scientific). We record all physiologic data using a custom‐designed LabView (NI, Austin, TX) program.

We ventilate for a total of 20 min—a 10‐min acclimation period followed by a 10 min nebulization period. To prevent condensation of the nebulized solution in the circuit, which could cause occlusion, we turn the nebulizer on/off during alternate 1‐min periods. At the end of the 10‐min nebulization period, we euthanize the rat (1 mL/kg IV of 300 mg/mL KCl in NS). We close the tracheal stopcock at PEEP, perform a thoracotomy, and excise the heart and lungs. We place the heart and lungs on the stage of an upright fluorescent confocal microscope (SP5, Leica Microsystems, Deerfield, IL), keep the lungs moist with a saline drip, connect the lungs to house air without allowing the lungs to deflate, inflate the lungs to 30 cmH_2_O, and then deflate the lungs to 10 cmH_2_O (Kharge et al., 2014). We capture fluorescent images in six standard locations (imaging details below).

#### 2.1.2 In vivo nebulization in LPS‐instilled rats

We anesthetize a rat (3.5% isoflurane in 100% oxygen), instill LPS (1 mL/kg of 0.75 mg LPS/mL NS; L2880, serotype O55:B5, MilliporeSigma) in the trachea (Silva et al., 2013), allow the rat to recover from anesthesia, wait 24 h, and follow the in vivo protocol of section 2.1.1, above. We capture fluorescent images in the six standard locations. In one experiment, to characterize cells in which fluorescein is highly concentrated, suggesting that the cells are actively phagocytic, we additionally micropuncture an alveolus and microinfuse the dye rhodamine 6G (100 μM; 83697, Sigma‐Aldrich, St. Louis, MO). Rhodamine 6G labels mitochondria and is used as a leukocyte marker (Baatz et al., 1995).

**FIGURE 1:**
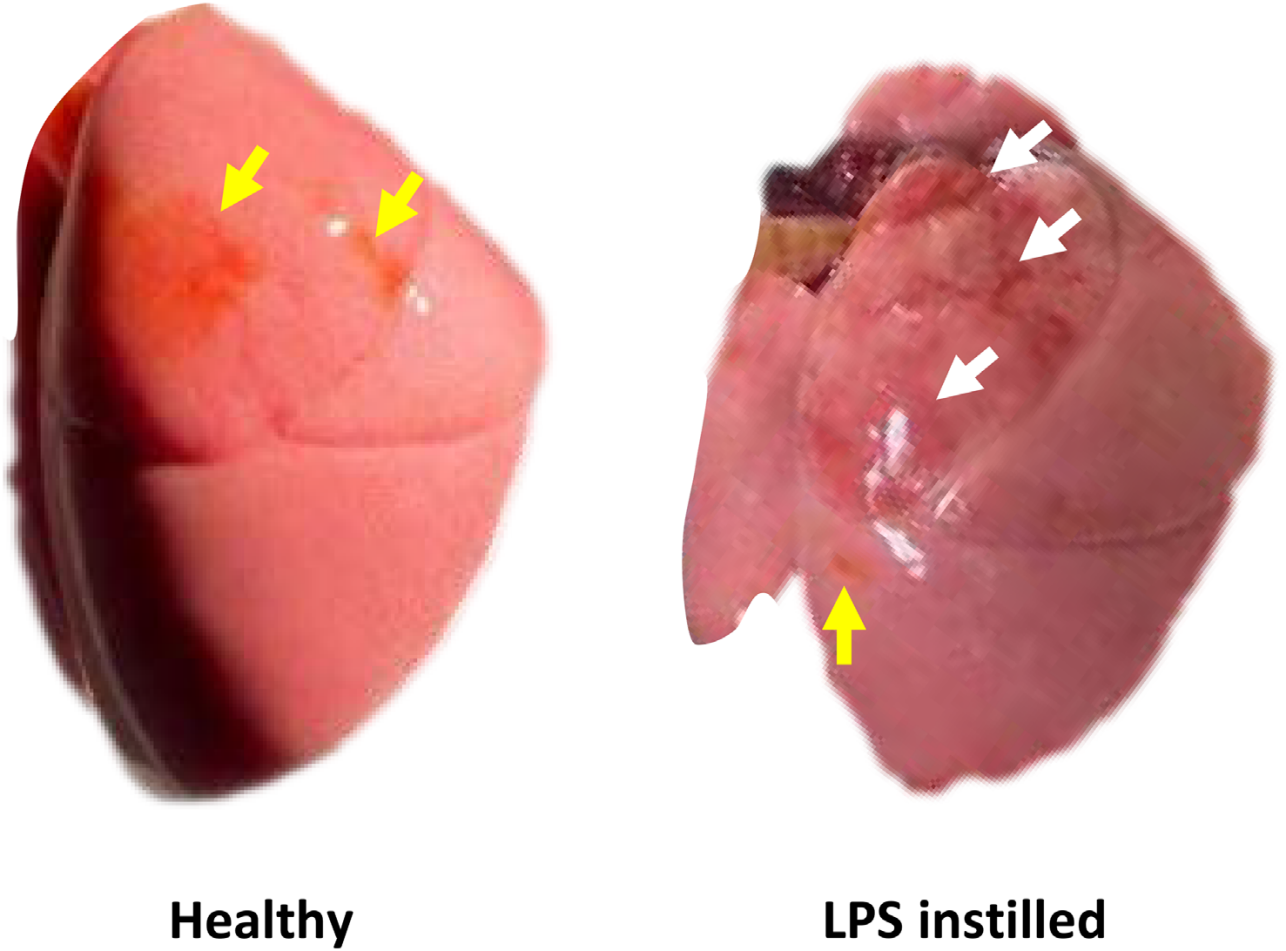
Gross appearance of lungs from rats nebulized with fluorescent solution. Compared with lungs from healthy rats, lungs from lipopolysaccharide (LPS)‐instilled rats exhibit patchy red, often collapsed, areas (white arrows) and are smaller in size. In lungs from healthy and LPS‐instilled rats, areas of concentrated fluorescein delivery are evident (yellow arrows).

In a separate series of experiments, instead of imaging in the six standard locations, we image injured regions. We select general imaging locations that are red and often collapsed, as evident by macroscopic inspection (white arrows of Figure 1, below). Then, by brightfield microscopy, we also use coloration to identify specific imaging regions. We capture brightfield and fluorescent images of injured regions and of one healthy‐looking region (Figure 5a, below) as detailed in section 2.2.2, below.

#### 2.1.3 Ex vivo nebulization in lungs from LPS‐instilled rats

We anesthetize a rat, instill LPS in the trachea, allow the rat to recover from anesthesia, wait 24 h, and follow the in vivo protocol of section 2.1.1. above, through the 10‐min acclimation period. Then, we euthanize the rat, close the tracheal stopcock at PEEP, excise the heart and lungs, connect the lungs to house air without allowing the lungs to deflate, and inflate the lungs to 10 cmH_2_O, which does not recruit the injured lungs. We identify injured regions for imaging as detailed for in vivo nebulization in LPS‐instilled rats in section 2.1.2, above. We mark the injured regions and one healthy‐looking region by microinjecting 1% Sudan‐black‐labeled castor oil into an adjacent subpleural alveolus and capture brightfield images of the marked regions. Next, we close the tracheal stopcock; connect the excised lungs, positioned as though the rat were in dorsal recumbency, to the ventilation circuit; start the ventilator using the same settings as in vivo; open the trachea to the ventilation circuit without allowing the lungs to deflate; and nebulize as in vivo. After nebulization, we close the tracheal stopcock at PEEP; connect the lungs to the house air without allowing the lungs to deflate; inflate the lungs to 30 cmH_2_O and deflate the lungs to 10 cmH_2_O; and capture fluorescent images of the marked injured and healthy‐looking regions.

#### 2.1.4 Healthy un‐nebulized controls

To determine background fluorescence intensity in the naïve rat lung, we anesthetize a rat, perform a tracheotomy, sacrifice the rat, and excise the lungs and image regions in upper, middle, and lower locations. To identify actively phagocytic cells in healthy, mechanically ventilated but un‐nebulized lungs, we follow the protocol of section 2.1.1, above, but omit nebulization. After lung excision, we microinject fluorescein solution into one middle and one lower location of the left lung and image the regions to identify, as we have previously with this protocol (Nguyen & Perlman, 2020), any actively phagocytic cells that may become evident. After phagocytic cells become evident, we microinject rhodamine 6G to assess leukocyte origin.

### 2.2 Lung imaging

To image the lungs, we hold a coverslip just in contact with the lung surface (Wu & Perlman, 2012). We level the cover slip using a new, custom‐built apparatus with orthogonal worm gears that enable adjustment of pitch and roll. We acquire images with LAS AF software (Leica Microsystems).

#### 2.2.1 Six standard locations

We capture fluorescent confocal images of regions in upper (cranial), middle, and lower (caudal) locations on the right and left side‐costal surfaces. In lungs from LPS‐instilled rats, we avoid imaging regions that we observe macroscopically (Figure 1, below) or by brightfield microscopy (Figures 5a and 6a, below) to have red or dark coloration, thus to be injured. Consequently, this series of images is one of relatively healthy regions, even in lungs from LPS‐instilled rats.

In each location, to capture long‐range spatial variation in fluorescence intensity, we first capture an ×2.5 (Fl Plan, 0.07 N.A., air) fluorescent image. By visual inspection of the low‐power region (see Figure 3a, below), we identify one of the darkest sub‐regions (which we term “dim”) and one of the brightest sub‐regions (“bright”). Then, we use the cover slip to support a water drop and obtain ×20 (Apo L, 0.5 N.A., water) images of the dim and bright sub‐regions. We excite the fluorescein at 488 nm (2% laser power) and collect emission between 495 and 700 nm with a HyD detector (gain 40). We capture Z‐stacks of images (1024 × 1024 pixels, 2‐μm optical section thickness, 3‐μm step size), from above the pleural surface to a subpleural depth of at least 30 μm. We randomize the side on which we start imaging and, after imaging the three regions on the first side, turn the lungs over and image the three regions on the second side. Total imaging time for each region and its two sub‐regions is ~20 min.

When we microinfuse rhodamine 6G, we collect fluorescein emission in a reduced range of 492–515 nm, excite rhodamine 6G at 543 nm and collect rhodamine 6G emission between 650 and 800 nm.

#### 2.2.2 Injured locations

We capture brightfield images with an ×10 (Pl Fluotar, 0.3 N.A., air) or ×20 objective and characterize the injury as moderate (moderate red coloration) or severe (dark coloration) as shown in Figures 5a and 6a, below. We also capture fluorescent confocal images, with the same settings as for the six standard locations.

### 2.3 Image analysis

We quantify fluorescein delivery as follows. We analyze 30‐μm subpleural images from ×20 Z‐stacks (ImageJ, NIH, Bethesda, MD). Even with our coverslip‐leveling mechanism, the cover slip is not perfectly parallel to the 2‐μm thick optical sections. As a result, there is typically a gradation in focus level across images that is apparent at the pleural surface. In the image at the pleural surface, we identify the line across the image that is focused at the pleural surface; subsequently, in the 30‐μm subpleural image, we draw an ROI around the brightest area of septal tissue along the same line. We use median gray level within the ROI to represent intensity of the sub‐region image. Further, we visually inspect brightened versions of all standard‐location images and characterize each sub‐region as aerated, having a few flooded alveoli, or having prominent flooding.

### 2.4 Statistical analysis

We assess statistical differences by multiple linear regression, accepting differences at *p* < 0.05.

## 3 RESULTS

We find that nebulized solution is delivered to the parenchyma at a uniform low basal level and to focal regions at higher level. In standard locations, there is significant intra‐ and inter‐region heterogeneity and actively phagocytic cells are observed. In severely‐injured regions, nebulized solution delivery is very low.

### 3.1 Nebulizer operation

Intermittent operation of the nebulizer prevents condensation from occluding the ventilation circuit. During the 10 min of intermittent nebulization, based on *n* = 3 in vitro trials with a mock lung connected to the ventilation circuit, 1.58 ± 0.11 (SD) mL of solution is aerosolized. Of that volume, 13.4 ± 1.8% exits the circuit and enters the lungs.

### 3.2 Physiologic data

Administration of LPS causes body weight to decrease by 3.6 ± 4.7% (*n* = 11, *p* < 0.05) over 24 h. Lungs from LPS‐instilled rats have patchy red coloration and a smaller size than healthy lungs (Figure 1). Macroscopic surface injury is upper‐lobe predominant in 86% of LPS‐instilled lungs. Injured lungs are readily distinguished from healthy lungs (91%–100% accurate identification from photographs by blinded authors C.S. and C.E.P.).

**FIGURE 2:**
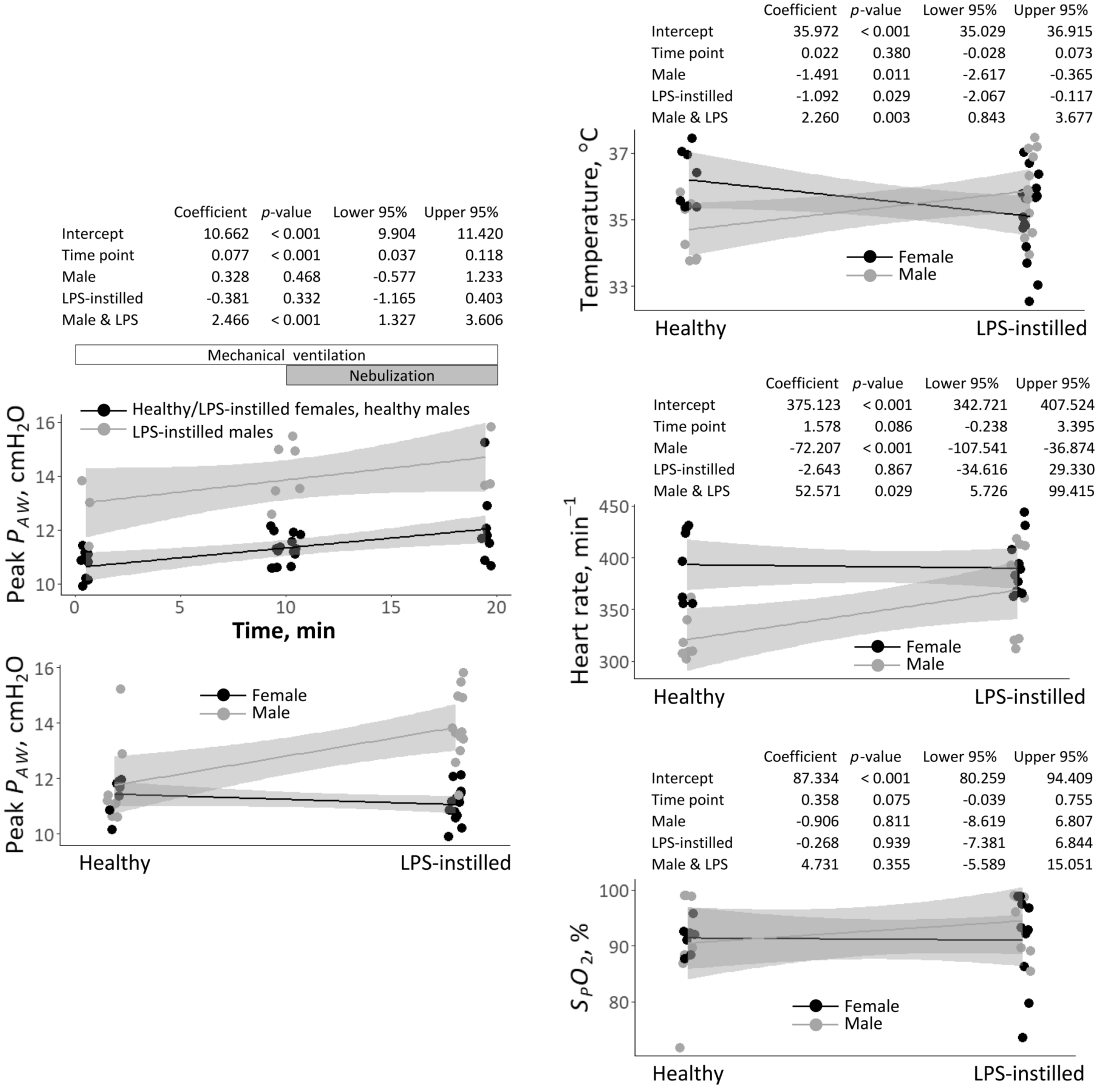
Physiologic data from in vivo nebulization in healthy and LPS‐instilled rats. Regression models with 95% confidence intervals plotted over scatter plots of raw data. Statistical analysis presented in charts above corresponding plots. For plot of peak airway entrance pressure, *P_AW_*, versus time, data are averages over four 1‐min periods—first and last minutes of 10‐min acclimation period preceding nebulization and first and last minutes of 10‐min nebulization period. Abbreviation: *S_P_O_2_*, peripheral arterial oxygen saturation.

Physiologic data from in vivo nebulization experiments are shown in Figure 2. Peak *P*_*AW*_ increases over time during mechanical ventilation. And the data for peak *P*_*AW*_, temperature, and heart rate show an interaction effect between sex and LPS‐induced injury. The results for peak *P*_*AW*_ suggest that duration of mechanical ventilation and LPS instillation in males may reduce lung compliance or increase airway resistance. In LPS‐instillation experiments, peak *P*_*AW*_ is 22.4 ± 2.7 cmH_2_O during ex vivo nebulization compared with 12.5 ± 1.7 cmH_2_O during in vivo nebulization (*n* = 4 and 7/group, respectively, *p* < 0.001). As lung excision should increase respiratory system compliance, this excision‐induced near‐doubling of peak *P*_*AW*_ indicates an increase in airway resistance. Lung‐excision‐induced increase in airway resistance has previously been attributed to airway collapse (Santini et al., 2021). In contrast with that prior study, however, we do not allow the lungs to deflate fully.

### 3.3 Methodologic considerations for imaging

**FIGURE 3:**
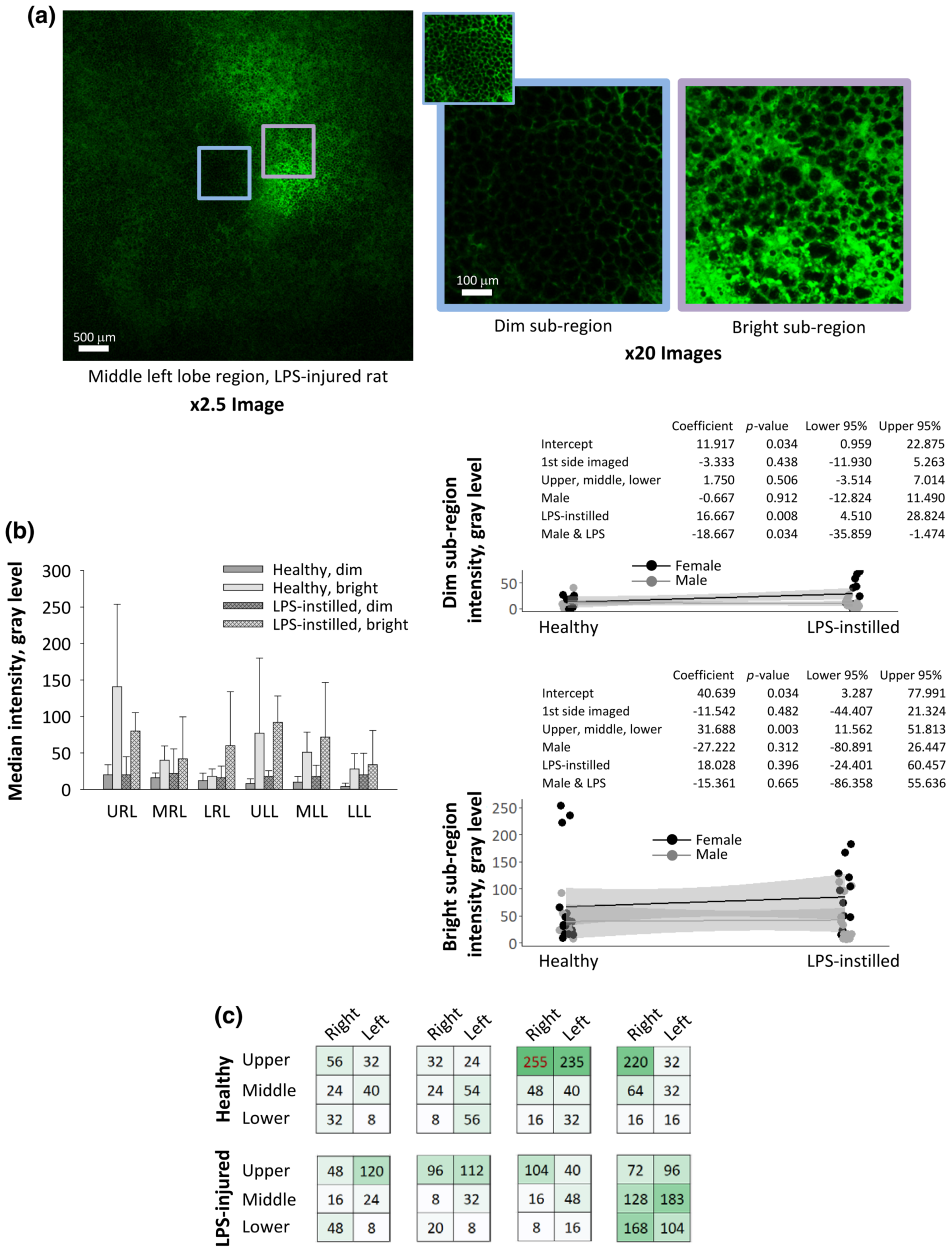
Fluorescein intensity in standard imaging locations following in vivo nebulization. (a) Representative images of a full region imaged at low power and of dim and bright sub‐regions imaged at high power. Dim and bright sub‐regions have median intensities of 40 and 183 gray levels, respectively. Main images are un‐brightened; inset of dim sub‐region is brightened to show presence of fluorescein. (b) Group data for six standard imaging locations—on side costal surfaces of upper (cranial) right lung, URL, middle right lung, MRL, lower (caudal) right lung, LRL, upper left lung, ULL, middle left lung, MLL, and lower left lung, LLL—plotted as mean ± standard deviation in bar chart. Data for dim and bright sub‐regions re‐plotted, separately, on right to show differences due to sex and LPS instillation. Statistical analysis performed separately for dim and bright sub‐regions. (c) Fluorescein intensity values for bright sub‐regions across six standard imaging locations of all eight lungs. Green shading is proportionate to intensity level. Median intensity is saturated (255 gray levels) in one sub‐region.

Whether a region is on the first‐ or second‐imaged side of the lungs does not affect degree of alveolar flooding (blinded analysis of standard‐location sub‐region images by authors C.S. and C.E.P.). Thus during imaging of the first side by upright microscopy, while the second side is dependent, there is not drainage to the second side. Further, the heterogeneous distribution of imaged fluorescence is attributable to heterogeneous fluorescein distribution and is not an optical artifact. Figure 3a shows dim and bright sub‐regions that are imaged sequentially with the same microscope settings and with the same coverslip held in the same orientation. Comparison of the brightened version of the dim sub‐region image and the bright sub‐region image demonstrates distinct fluorescence patterns.

### 3.4 Fluorescein in six standard locations of healthy/LPS‐instilled lungs, following in vivo nebulization

Following in vivo nebulization in healthy and LPS‐instilled rats, fluorescein reaches most regions (Figure 3a,b). Compared with un‐nebulized control lungs (median gray level of 0), we detect fluorescein (median gray level >0) in 83 and 100% of dim and bright sub‐regions, respectively, in the six standard imaging locations. Dim sub‐region intensity does not vary across imaging locations. With LPS‐instillation, however, dim sub‐region intensity increases in females but not males (Figure 3b). Across healthy and LPS‐instilled lungs, bright sub‐region intensity is greatest in upper locations and decreases toward lower locations (Figure 3b). Consequently, bright sub‐region intensity in upper, middle, and lower locations is 6.4 ± 4.9, 3.3 ± 3.0, and 2.3 ± 2.8 times greater, respectively, than average dim sub‐region intensity across all locations. The highest bright sub‐region intensity is in an upper location in six of eight lungs (Figure 3c).

### 3.5 Initiation of inflammation observed in standard imaging locations

**FIGURE 4:**
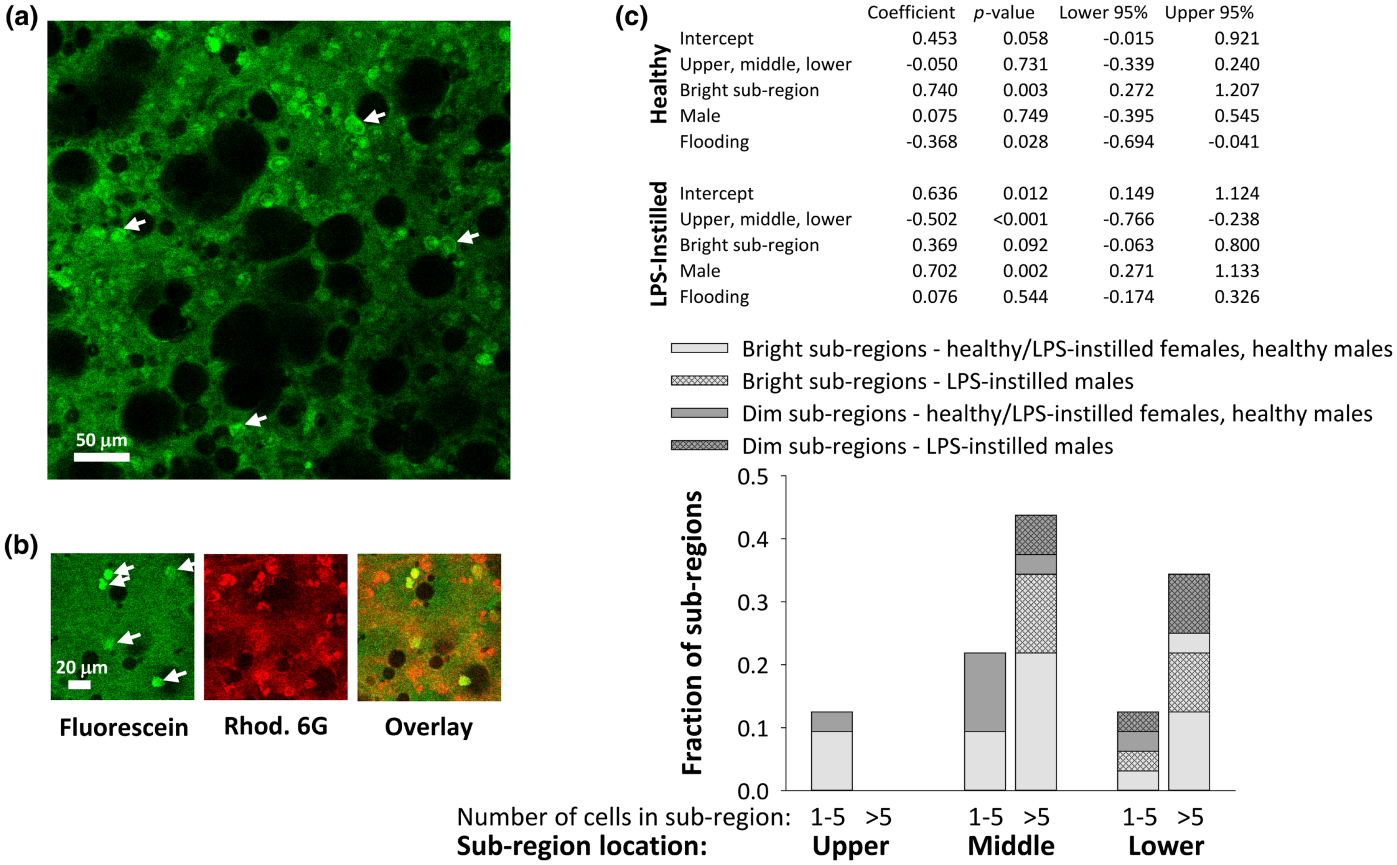
Activate phagocytes present in standard imaging locations following in vivo nebulization. (a) Representative region in which bright green cells (e.g., arrows) are evident. Image is from 12‐μm subpleural plane in middle right lobe of LPS‐instilled lungs. Cells are most apparent in high planes near pleural surface. (b) Cells are of leukocyte origin. Rhodamine 6G labels all green cells. (c) Histogram showing percentages of dim and bright sub‐regions in indicated lung locations containing a few (1–5) or many (>5) cells.

In some imaged regions, bright green cells are evident (Figure 4a). The cells are visible because they are brighter than the surrounding alveolar liquid, suggesting that they are actively phagocytosing, and thereby concentrating, fluorescein. Further, 63% of the bright green cells are round, suggesting that many may be macrophages. We find that rhodamine 6G labels 100% of the bright green cells, indicating that they are likely of leukocyte origin (Figure 4b). Rhodamine 6G also labels other cells that are likely alveolar epithelial type II cells, which have a high mitochondrial density (Ruaro et al., 2021).

In control lungs from healthy rats that are mechanically ventilated but not nebulized, active phagocytes are observed in the two imaged locations. In standard locations of ventilated and nebulized lungs, active phagocytes are evident, to the same degree, in healthy and LPS‐instilled lungs (Figure 4c). In healthy lungs, active phagocytes are more often evident in bright than dim sub‐regions and in aerated than flooded sub‐regions. In LPS‐instilled lungs, active phagocytes are more often evident in lower than upper locations and in males than females.

### 3.6 Fluorescein in injured regions of LPS‐instilled lungs, following in vivo nebulization

**FIGURE 5:**
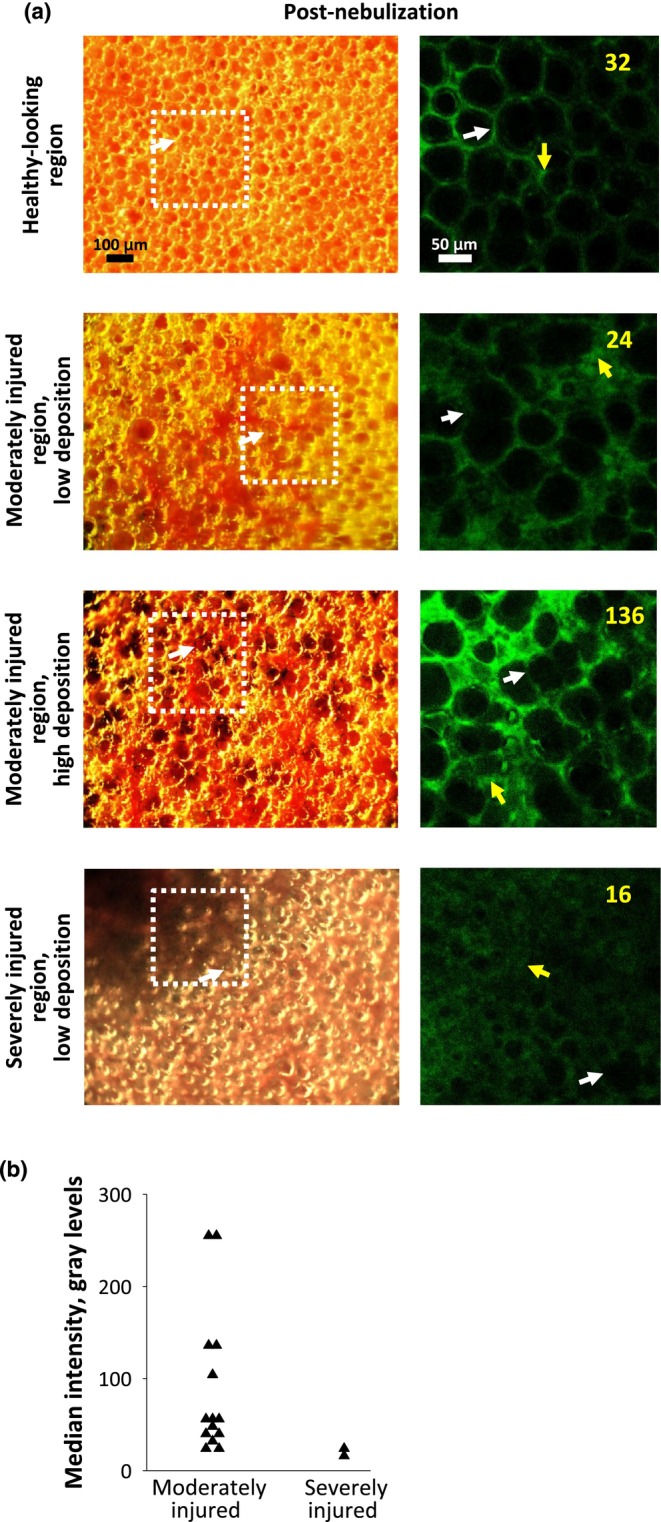
Fluorescein intensity in injured regions following LPS administration and in vivo nebulization. (a) Example pairs of brightfield (left) and fluorescent (right) images. White rectangles mark locations of fluorescent images. White arrows mark corresponding alveoli between images. In fluorescent images (all brightened 60%), yellow arrows mark locations of intensity determinations. Yellow text reports intensity values. Images of healthy‐looking region included as counter example, to demonstrate that LPS‐injured regions can be identified by coloration in brightfield images, but not included in analysis. (b) Quantification of fluorescence intensity in original, unadjusted fluorescent images from moderately and severely injured regions. Intensity is saturated in two moderately injured regions. Statistics not assessed as severely injured group contains only two data points.

For in vivo nebulization of LPS‐instilled lungs, fluorescein intensity in moderately injured regions can be low or high and fluorescein intensity in severely injured regions is low (Figure 5). With high fluorescein intensity observed in moderately‐injured regions imaged only after nebulization, it is not possible to determine whether already‐injured regions received high deposition or whether nebulization contributed to injury.

### 3.7 Fluorescein in injured regions of LPS‐instilled lungs, following ex vivo nebulization

**FIGURE 6:**
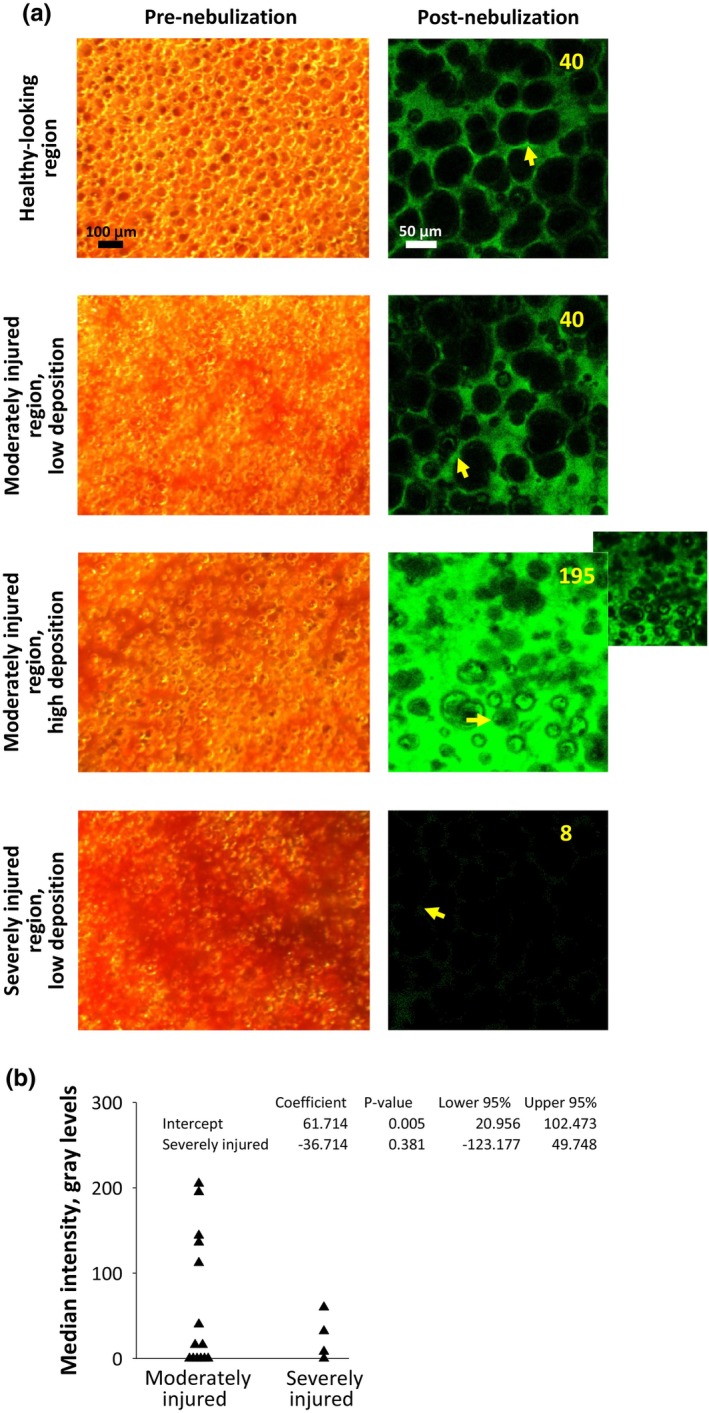
Fluorescein intensity in injured regions following LPS administration and ex vivo nebulization. (a) Example pairs of brightfield images captured before ex vivo nebulization (left) and fluorescent images of same regions captured after ex vivo nebulization (right). Due to nebulization, corresponding alveoli can not be identified between images. In fluorescent images (all brightened 60%), yellow arrows mark locations of intensity determinations. Yellow text reports intensity values. Images of healthy‐looking region included as counter example, to demonstrate that injured regions can be identified by coloration in brightfield images prior to mechanical ventilation/nebulization, but not included in analysis. For moderately injured region with high deposition, fluorescein not saturated in original, un‐brightened image (inset). (b) Quantification of fluorescence intensity in original, unadjusted fluorescent images from moderately and severely injured regions.

For ex vivo nebulization of LPS‐instilled lungs, we identify injured regions before nebulization and detect fluorescein after nebulization. We observe the same pattern as with in vivo nebulization. Fluorescein intensity in moderately injured regions can be low or high and fluorescein intensity in severely injured regions is low (Figure 6). Thus, already‐moderately‐injured regions can receive substantial delivery of nebulized solution. Already‐severely‐injured regions appear not to receive substantial delivery of nebulized solution.

## 4 DISCUSSION

We present here a quantitative analysis of the levels of nebulized solution that are received by different, often neighboring, regions of the lung parenchyma. And we present the first data, to our knowledge, on how lung injury affects nebulized solution delivery.

### 4.1 Relevance of nebulized volume

We nebulize an amount of fluorescein solution, 6.1 mL/kg, that enables us to visualize fluorescein in subpleural alveoli. Of this volume, 0.8 mL/kg passes beyond the tracheal cannula into the trachea and lungs; we do not quantify the volume delivered specifically to the parenchyma. The total nebulized volume is the same as that of surfactant delivered by nebulization to neonates (Cummings et al., 2020). Thus, while it is difficult to compare delivery across nebulization methods and Marangoni flow would be expected to assist delivery of surfactant to the parenchyma, the volume we deliver is clinically relevant.

### 4.2 Results from standard imaging locations

Almost all regions receive at least the low‐but‐detectable dim‐sub‐region level of deposition, which may be considered a basal level of delivery provided by nebulization (Figure 3b). But we observe heterogeneity in that focal regions receive high deposition. The focal high‐deposition regions are brighter in upper than lower locations. This result is likely attributable to there being fewer airway generations in upper than lower lobes. We analyze anatomical data that have been collected, by lobe, for all conducting airways of a rat lung. In the upper right lobe, average and maximum conducting airway generation numbers are 12.4 ± 3.7 and 23, respectively; in the lower right lobe, average and maximum conducting airway generation numbers are 18.6 ± 4.8 and 33, respectively (Raabe et al., 1976). Similarly in human lungs, there are fewer airway generations in upper than lower lobes (Everaerts et al., 2019). Thus, although we are limited to imaging surface alveoli, we image alveoli that are distal to the trachea by a wide range of path lengths. Deposition in interior alveoli of the lower lobes could reasonably be expected to be similar to that in surface alveoli of the upper lobes. In general, the pattern that we observe—relatively uniform basal deposition with focal bright regions that are more intense in alveoli arising from earlier airway generations—is likely to be representative of that across much of the parenchyma.

In dim sub‐regions of LPS‐instilled lungs, we find reduced fluorescein deposition in males compared with females (Figure 3b). This finding is consistent with *P*_*AW*_ being elevated in LPS‐instilled males compared with LPS‐instilled females (Figure 2).

We find active phagocytes present in standard imaging locations of healthy and LPS‐instilled lungs. In healthy lungs, more active phagocytes are evident in bright or aerated sub‐regions (Figure 4c). This result must be interpreted with caution. Some threshold level of fluorescein may need to be present in order for the cells to be able to concentrate a sufficient amount of the dye to become visible. Alternatively, too much fluorescein in regions that are flooded by nebulized solution might mask the presence of the cells. Thus, whether more active phagocytes are in fact present in bright or aerated regions remains to be determined.

In LPS‐instilled lungs, more active phagocytes are evident in lower locations and in males (Figure 4c). Intra‐tracheal LPS instillation has been shown to cause patchy injury and reduce compliance (Chen et al., 2016; Lima Trajano et al., 2011; Puntorieri et al., 2016). We too observe patchy injury, and we find the injury to be upper‐lobe predominant. We speculate that the lower lobe predominance of active phagocytes is due to LPS‐induced stiffening of upper lobes and a resultant increase in strain in lower lobes (Gattinoni & Pesenti, 2005). The disproportionate number of active phagocytes in LPS‐instilled males may be due to the deleterious effect of LPS on lung mechanics in males (Figure 2).

### 4.3 Results from injured locations

Our findings suggest that, as would be expected, injury can affect the deposition pattern of nebulized solution. In imaging evidently injured regions in lungs from LPS‐instilled rats, we find that moderately injured regions can receive high fluorescein deposition but severely injured regions receive negligible deposition (Figures 5 and 6). The latter is presumably attributable to more severely injured regions having lower compliance.

Fluorescein intensity in injured regions is comparable between in vivo and ex vivo nebulization (Figures 5b and 6b), yet fluorescein intensity in injured regions could potentially be altered by multiple factors. Fluorescein could be quenched by hemoglobin in hemorrhagic regions. Any hemorrhage induced by LPS instillation, however, should be comparable between the in vivo/ex vivo nebulization protocols. Following in vivo nebulization, fluorescein that diffuses to the vasculature could be washed away. In ex vivo nebulization, higher airway resistance could reduce fluorescein delivery to the parenchyma. Whether comparable fluorescein intensity in in vivo and ex vivo experiments reflects comparable fluorescein delivery is uncertain. A different approach would be needed to resolve this uncertainty.

### 4.4 Sex‐LPS interaction effects

We find that LPS instillation increases *P*_*AW*_, reduces dim sub‐region fluorescein intensity, and increases actively phagocytic cells in males relative to females (Figures 2, 3b, and 4c). We attribute these findings to altered mechanics, with the *P*_*AW*_ data suggesting that LPS reduces lung compliance or increases airway resistance in male but not female rats. These results contribute to a small but growing literature on sex‐based differences in inflammatory effects on lung mechanics, but one in which trends have yet to emerge. Voltz et al. (2008) found bleomyecin to reduce compliance more in males than females. In contrast, Puntorieri et al. (2016) found LPS to reduce compliance significantly only in females, not males, and Boylen et al. (2011) found diesel exhaust particles to reduce compliance significantly only in females, not males. That we observe an opposite trend with LPS than Puntorieri et al. might be explained by differences in LPS serotype, LPS dose, experimental time course or model species between the studies, and requires further investigation.

### 4.5 Limitations

Limitations of our study are as follows. We quantify variability in levels of fluorescein between imaged regions, but not the extent of lung areas that receive each level of fluorescein. We test one nebulizer model with one set of ventilation parameters and our results may not be generalizable. Parenchymal delivery of nebulized solution should be greater with jet nebulization, the other principal form of nebulization, as jet nebulizers create smaller, ~2 μm aerosol particles that more easily penetrate to the periphery of the lungs. Given the small size of murine lungs, clinical relevance is uncertain. Heterogeneity of distribution is expected to be greater in larger human lungs.

## 5 CONCLUSIONS

We find that nebulization delivers a relatively uniform basal level of solution across the parenchyma excepting to severely injured regions. Further, there is heterogeneity within and between locations. The greatest heterogeneity is in upper locations where average bright sub‐region intensity is 600% that of dim sub‐regions. Nebulization could be problematic in certain scenarios—for example if a drug had a low toxicity threshold, needed to be delivered uniformly across the parenchyma, needed to be delivered at high concentration across the parenchyma or needed to be delivered to severely injured regions. The level and heterogeneity of deposition should be considered in the context of a particular condition and drug when assessing the benefits and risks of delivering a therapeutic via nebulization. For example, due to the lack of delivery to severely injured regions, nebulization might not be appropriate for delivering certain therapeutics for lung injury. For many conditions and drugs, however, the observed basal level of deposition and heterogeneity are likely to be acceptable.

## FUNDING INFORMATION

Funded by NIH R01/R56 HL113577.

## CONFLICT OF INTEREST STATEMENT

The authors have no conflicts to disclose.

## ETHICS STATEMENT

All protocols in this study were approved by the Stevens Institute of Technology Institutional Animal Care and Use Committee.

## Data Availability

The data that support the findings of this study are available from the corresponding author upon reasonable request.
